# Oxidative Stress and Necrotizing Enterocolitis: Pathogenetic Mechanisms, Opportunities for Intervention, and Role of Human Milk

**DOI:** 10.1155/2018/7397659

**Published:** 2018-07-02

**Authors:** Arianna Aceti, Isadora Beghetti, Silvia Martini, Giacomo Faldella, Luigi Corvaglia

**Affiliations:** Neonatal Intensive Care Unit, Department of Medical and Surgical Sciences, S.Orsola-Malpighi Hospital, University of Bologna, Bologna, Italy

## Abstract

This review will examine the role of oxidative stress (OS) in the pathogenesis of necrotizing enterocolitis (NEC) and explore potential preventive and therapeutic antioxidant strategies. Preterm infants are particularly exposed to OS as a result of several perinatal stimuli and constitutive defective antioxidant defenses. For this reason, OS damage represents a contributing factor to several complications of prematurity, including necrotizing enterocolitis (NEC). Being NEC a multifactorial disease, OS may act as downstream component of the pathogenetic cascade. To counteract OS in preterm infants with NEC, several antioxidant strategies have been proposed and different antioxidant compounds have been experimented. It is well known that human milk (HM) is an important source of antioxidants. At the same time, the role of an exclusive HM diet is well recognized in the prevention of NEC. However, donor HM (DHM) processing may impair antioxidant properties. As DHM is becoming a common nutritional intervention for high risk PI, the antioxidant status of preterm and DHM and potential ways to preserve its antioxidant capacity may merit further investigation.

## 1. Introduction

Necrotizing enterocolitis (NEC) is the most devastating gastrointestinal neonatal disease [1]. NEC occurs at a frequency of 1–3 per 1000 live birth, and almost 90% of the cases affect infants born preterm, the risk being inversely related to birth weight and gestational age. Despite advances in perinatal and neonatal care, NEC remains a leading cause of morbidity and mortality in preterm infants, with mortality rates reaching approximately 30% [2].

NEC is a multifactorial disease with a poorly understood pathogenesis. Prematurity itself is recognized as the main risk factor; intestinal immaturity, imbalanced microvascular tone, abnormal microbial intestinal colonization, and dysregulated immune response, together with hypoxic-ischemic injury, lead to intestinal inflammation and necrosis [3]. Due to NEC multifactorial nature and frequently abrupt onset, univocal treatment and management of the disease are still debated.

Newborns and especially preterm infants are more exposed to oxidative stress (OS) than adults and children. Reactive oxygen species (ROS) damage seems to play a role in many neonatal diseases. In 1988, Saugstad proposed the concept of “oxygen radical diseases of neonatology,” including respiratory distress syndrome, bronchopulmonary dysplasia, periventricular leukomalacia, patency of the ductus arteriosus, retinopathy of prematurity (ROP), and NEC.

This article will review the role of OS in the pathogenesis of NEC and will explore potential preventive and therapeutic antioxidant strategies, with a focus on strategies for reducing OS by implementing human milk (HM) feeding.

A narrative review of published studies reporting the role of OS in the pathogenesis of NEC was performed. The literature search was run in PubMed and Embase. The PubMed string was built up by combining all the terms related to OS and NEC, using PubMed MeSH terms, free-text words, and their combinations through the most proper Boolean operators, in order to be as comprehensive as possible. The same criteria were used for searching Embase. An additional search was performed by including HM among search criteria. The reference lists of relevant studies were searched for additional papers to be included.

## 2. Oxidative Stress in Neonatology

ROS and nitrogen reactive species (RNS), which are collectively referred to as ROS, are normally produced in living organisms. Biologically, significant ROS elements include hydroxyl (OH^•^), superoxide (O^−2•^), nitric oxide (NO^•^), hydrogen peroxide (H_2_O_2_), ozone (O_3_), singlet oxygen (^1^O_2_), organic peroxides (ROOH), and peroxynitrite (ONOO^−^). Reactive iron species are also included into ROS [4]. When produced in physiological concentrations, ROS behave as important mediators of almost all cellular functions, such as energy production and immune response. When produced in excess, they induce OS, which is responsible for tissue and cellular injury consisting in peroxidation of membrane lipids, alterations in protein function and structure, and DNA damaging.

ROS are generated via various mechanisms such as hypoxia, ischemia-reperfusion, hyperoxygenation, neutrophil and macrophage activation, mitochondrial dysfunction, endothelial cellular damage, and prostaglandin metabolism. ROS are neutralized by antioxidant systems which include endogenous and exogenous, enzymatic and nonenzymatic, and vitamin and nonvitamin components. Currently, known endogenous antioxidants include superoxide dismutase (SOD), catalase, glutathione peroxidase, glutathione reductase, glutathione (GSH), flavonoids, bilirubin, uric acid, melatonin, endogenous organic selenium, and the metal-binding proteins transferrin, ferritin, lactoferrin, ceruloplasmin, and albumin. Exogenous antioxidants include vitamin C, vitamin E, carotenoids, phenolic acids, flavonoids, acetylcysteine, exogenous selenium, zinc, magnesium, and copper.

There is a delicate balance between free radicals and antioxidants. OS occurs when ROS production overcomes antioxidant capacity or alternatively, when the antioxidant system is defective. OS is involved in several natural phenomena, from birth to aging, starting from pregnancy and continuing during postnatal life [5]. Birth represents a significant oxidative challenge, because of the shift from the relative low-oxygen intrauterine environment to the high-oxygen extrauterine environment [6]. The fetal antioxidant system is upregulated in the last 15% of gestation in preparation for the transition to extrauterine life. During this critical time window, nonenzymatic antioxidants cross the placenta in increasing concentrations and endogenous fetal antioxidant enzyme, such as SOD, has been shown to increase by 150% [7]. Thus, newborns are exposed to OS during transition to neonatal life and labor; several studies have documented high levels of OS markers in mothers and healthy term infants immediately after birth, further increasing during the first few days of neonatal life [8].

Human studies have demonstrated that increased ROS production occurs in preterm infants compared to term infants and is associated with a relative lack of antioxidant enzyme concentration and activity. Furthermore, Sandal et al. [9] have shown that in preterm infants and term small-for-gestational age (SGA) infants, total oxidant stress measured on cord blood is higher and total antioxidant capacity is lower than in term appropriate-for-gestational age (AGA) infants.

Preterm infants are especially exposed to ROS damage for several reasons. Birth oxidative challenge may be even more difficult for preterm infants, because preterm birth interrupts the normal developmental upregulation of the antioxidant system. Moreover, the ability of preterm infants to increase antioxidant production in response to oxidant stimuli is impaired [7]. On the other hand, increased ROS production occurs in preterm infants; actually, early after birth they are often exposed to high oxygen concentration due to resuscitation and O_2_ supplementation. In addition, they have particularly high metabolic turnover rates and are more susceptible to infection, because of hospitalization and relative immunodeficiency. PI also have higher levels of free iron than term infants, which can lead to the production of the highly toxic OH^•^ [4, 6]. Last but not least, nutritional factors contribute to ROS exposure. In preterm infants who cannot receive enteral feeding, total parenteral nutrition (TPN) is critical to provide essential nutrients. However, TPN solutions are often contaminated with oxidation product [10]. Even when enteral nutrition is introduced, mother milk availability is often low and formula milk is used instead. HM is known to be a better scavenger of free radicals than infant formula [11]. Studies have demonstrated that dietary factors may contribute to the overall level of oxidative stress in preterm infants; specifically, preterm infants fed with HM have shown less evidence of oxidative stress after birth than those who were formula fed [12].

Normal pregnancy is associated with enhanced ROS production due to a high metabolic rate and oxygen requirements. The maintenance of pregnancy and fetal development depends on oxygen supply and ROS neutralization; moreover, in complicated pregnancy OS increases. Whenever OS occurs, it may cause abnormal placentation, endothelial dysfunction, and abnormal fetal growth, and it has been associated to several pregnancy disorders including preeclampsia, fetal growth restriction, and preterm birth [5]. OS markers have been measured in cord blood of preterm infants as an indicator of perinatal OS. They have been associated with increased risk for “free radical-related diseases,” suggesting that in utero/perinatal OS is a significant risk factor, especially in premature newborns [13].

Several investigators examined the relationship between the oxidative state of the mother and the newborn at birth. A positive correlation was found between the oxidative status of healthy mothers and that of term infants measured in venous maternal and umbilical cord blood, respectively, with higher maternal OS correlating with an even higher OS of the newborn [14]. Other studies have investigated the relation of different OS markers and lipid-soluble micronutrients, in cord plasma and maternal serum, with growth retardation and prematurity, showing contrasting results [15].

## 3. The Role of Oxidative Stress in the Pathogenesis of NEC

Several studies have suggested a role of OS in the pathogenesis of NEC; Aydemir et al. [16] compared, in preterm infants with and without NEC, the global oxidant/antioxidant status, by measuring total antioxidant capacity (TAC), total oxidant status (TOS), and oxidative stress index (OSI). Infants with NEC had significantly higher TOS and OSI levels compared with controls, higher levels of TOS and OSI being associated with the severity of NEC. Furthermore, Perrone et al. [17] demonstrated a strong association between the concentration of OS markers in cord blood and the occurrence of NEC in preterm infants.

According to epidemiologic observations and studies performed in animal models, the pathogenesis of NEC is thought to be multifactorial and largely related to the immaturity of the gastrointestinal tract [3]. The intestinal mucosa of preterm infants is exposed to constant injury, due to perinatal insults such as hypoxia, hypothermia, and formula feeding. Innate immune receptor Toll-like receptor 4 (TLR4) seems to have a central role in NEC pathogenesis. Excessive signaling in the epithelial TLR4 pathway in response to lipopolysaccharide (LPS) presented by Gram-negative bacteria leads to the loss of enterocytes through apoptosis, followed by delayed repair through inhibition of migration and TLR4-mediated loss of intestinal stem cells [18]. These factors lead to the translocation of bacteria and LPS into the circulation and consequently to proinflammatory cytokine production, ROS production, increased expression of inducible nitric oxide synthase (iNOS), and impaired perfusion via loss and dysregulation of endothelial nitric oxide synthase (eNOS) mediated by TLR4 [19].

Experimental models have provided clues of a direct link between ROS production in the premature gut and NEC. OS may act as a downstream component in the inflammatory cascade which results in intestinal injury and even as a concomitant cause. Nitric oxide seems to play a crucial and ambiguous role in OS-related NEC damage. NO is produced from arginine in a reaction catalyzed in the intestine mainly by two NO synthases (NOS), eNOS and iNOS. eNOS is constitutively expressed in the intestinal capillaries, and low concentrations of NO are responsible for the regulation of vascular tone. iNOS is mainly located in immune cells and activated by proinflammatory cytokines during inflammation and pathogen response. Sustained upregulation of iNOS in the intestinal mucosa is known to occur in preterm infants during the development of NEC [20]. In a neonatal rat model of NEC, increased concentration of iNOS caused by LPS was found in the intestinal mesentery in the late stage of the disease [21]. This upregulation may contribute to intestinal injury via high levels of NO. NO readily reacts with the O^−2•^ to form peroxynitrite, a reactive oxygen and nitrogen species that is highly toxic to epithelial cells [22]. eNOS is involved as well [23]. During OS, eNOS switches from producing NO to O^−2•^ [24]. This switch in enzyme function is called “eNOS uncoupling.” Once generated, O^−2•^ can form additional ROS, exaggerating the uncoupling. Studies on rat models indicate that NOS uncoupling becomes worse during disease progression [21].

It has also been proposed that the underlying initial condition in NEC pathogenesis is the reduced ability of the neonatal gut epithelial cells (NGECs) to clear OS when exposed to enteral feeding [25]. An agent-based computational model has demonstrated that impaired OS management can lead to apoptosis and inflammation of NGECs when additional bacterial TLR4 activation occurs.

## 4. Potential Antioxidant Strategies for Preventing NEC

Given the role of OS in the pathogenesis of NEC and the relatively deficient oxidant/antioxidant balance in preterm infants, several studies have investigated the potential protective role of direct and indirect antioxidant strategies; recently, astragaloside IV (AS-IV), a flavonoid, has been found to be effective in the protection of NEC-induced ileum degeneration via the regulation of the vitamin D3-upregulated protein 1/nuclear factor- (NF-) *κ*B signaling pathway [26]. AS-IV has also been shown to decrease the levels of malonyldialdehyde (MDA), one of the products of lipid oxidation, and to improve the levels of GSH and SOD in rats with experimental NEC. In addition, AS-IV inhibited NEC-induced elevation of proinflammatory mediators, such as interleukin- (IL-) 6, IL-1*β*, tumor necrosis factor- (TNF-) *α*, and NF-*κ*B. Similarly, Ozdemir et al. [27] have suggested that all-*trans*-retinoic acid (ATRA), an active and natural derivative of vitamin A, has a protective effect on intestinal injury through its anti-inflammatory and antioxidant properties. Rats with NEC injected with ATRA showed lower intestinal MDA and TNF-*α* levels and higher SOD and glutathione peroxidase activities than controls. Yazc et al. [28] have found that boric acid and 2-aminoethoxydiphenyl borate partly prevent the occurrence of NEC, by decreasing GSH consumption and enhancing the antioxidant defense mechanism. Sheng et al. [29] have shown that hydrogen-rich saline reduced the incidence and severity of NEC in a neonatal rat model, by inhibiting mRNA expression of proinflammatory mediators, downregulating lipid peroxidation, and enhancing total antioxidant capacity. According to Ozdemir et al. [30], N-acetylcysteine (NAC) also has a protective effect on intestinal injury, through its anti-inflammatory and antioxidant properties. NEC rats treated with NAC had lower intestinal MDA levels and higher antioxidant enzyme activity; NAC also reduced intestinal TNF-*α* concentration. In vitro studies [31] suggested that hydrogen sulfide successfully rescues epithelial cell damage induced by OS in rat intestinal cells. Karatepe et al. [32] showed that the overexpression of the vascular endothelial growth factor in an experimental NEC model enhanced angiogenesis, alleviated villous atrophy and tissue edema, and was linked to reduced inflammation, apoptosis, and OS markers.

## 5. Exploring the “Milky Way”: Reducing Oxidative Stress by Implementing HM Feeding

It is well known that the characteristics of enteral feeding have a strong impact on the risk of NEC in preterm infants; specifically, it is generally recognized that the risk of NEC is reduced when preterm infants are fed an exclusively HM-based diet compared to diets containing bovine-derived products (formula and/or traditional HM fortifiers) [33]; the detrimental effect of bovine-based products appears to be related to an increased intestinal permeability, a direct toxic effect to the gut epithelial cells, and also to an upregulation of OS [34].

Friel et al. [35] demonstrated that HM, provided by mothers of both term and preterm infants, has better antioxidant properties than formula and that preterm and term HM have equal resistance to OS. The same authors also examined the effect of HM fortification on markers of OS in preterm infants and found that infants fed with HM plus HM fortifier had the highest urinary levels of F2-isoprostanes, compared to both infants fed with exclusive HM and formula [12]. This finding, which has not been further explored, might explain, at least partially, the beneficial effect of HM diets without any bovine-derived supplement on NEC incidence in preterm infants. Sandal et al. [9] investigated antioxidant properties of preterm and term HM, showing no difference in milk oxidant status between mothers of term and preterm infants. According to these data and given the relationship between OS and NEC, ad hoc strategies aimed at implementing exclusive HM feeding in infants at risk for NEC appear to be of paramount importance.

When own mother's milk (OMM) is unavailable, donor HM (DHM) provided by a HM bank is considered the best alternative for feeding preterm infants [36], and recent data have suggested that DHM feeding is linked to a beneficial effect in terms of NEC reduction [37].

Preparation, pasteurization, and distribution of DHM are regulated by specific recommendations [38–41]. The Holder pasteurization method is universally recommended for HM banks, as it guarantees the best compromise between microbiological safety and preservation of HM bioactive components [42]. Pasteurization is known to impair several nutritional and functional components of HM, such as immunoglobulin and lactoferrin [43]. Furthermore, Holder pasteurization, which is the pasteurization method commonly used in HM banks, appears to impact on the antioxidant capacity of DHM [44, 45] and to affect also selected antioxidant compounds which are present in HM, including GSH [46] and vitamin D [47]. Hanson et al. [11] recently compared several antioxidant components, including *α*-carotene, *β*-carotene, lycopene, lutein + zeaxanthin, retinol, and *α*- and *γ*-tocopherol, between preterm and donor HM; the authors documented lower levels of all the examined components in DHM compared to those in preterm HM.

The use of DHM has become common practice in many neonatal units, when OMM is insufficient or unavailable. However, since experimental data suggest that DHM processing negatively affects many functional properties of HM, it seems reasonable to explore novel treatments for DHM, aimed at preserving as many functional properties as possible [38], including antioxidant components [48]. In this perspective, growing literature is exploring the role of pasteurization methods alternative to Holder in preserving HM bioactive components, such as functional proteins [49] and fatty acids [50]. However, at present, no data about the effect of pasteurization methods other than Holder on OS markers are available.

For this reason, further research should be targeted to specifically explore the effect of DHM processing on HM antioxidant status. Whether HM antioxidant properties would result to be impaired by HM processing, it would be reasonable to explore if exogenous supplementation of antioxidant compounds would help in restoring these properties, thus preventing NEC occurrence or reducing NEC severity. In this perspective, current literature about specific targets for further research is now explored.

### 5.1. Glutamine and Arginine

In an experimental neonatal rat model of NEC, enteral supplementation with glutamine and arginine has been shown to exert a favorable effect on lipid peroxidation and antioxidant enzyme levels in the small intestine [51], probably through an increased production of NO. A recent systematic review of published randomized controlled trials (RCTs) [52] suggested L-arginine supplementation to be protective against NEC without any effect on neurodevelopmental outcomes at 3 years correct age. Although promising, these results were based on data from only two trials including 235 preterm infants, thus needing to be confirmed on a larger scale.

### 5.2. Carotenoids

As previously shown, carotenoids are antioxidant compounds which are present in preterm HM and whose concentration is severely impaired by pasteurization [11]. A multicenter RCT was conducted in order to examine the role of lutein and zeaxanthin enteral supplementation on the incidence of OS-related diseases in preterm infants [53]; carotenoid supplementation was related with a nonsignificant decrease in the incidence of NEC and ROP, and with a 50% decrease in the rate of progression from early to threshold ROP.

### 5.3. Melatonin

Melatonin is excreted in term and preterm HM in similar amounts; interestingly, melatonin, similarly to other antioxidant compounds, is excreted in preterm HM following a circadian rhythm [54]. Several studies have focused on the role of melatonin in counteracting oxidative injury in newborns; melatonin has been used in cases of asphyxia, respiratory distress syndrome, and sepsis, with no report of any significant side effect [5]. Data about the potential use of melatonin for NEC are limited to neonatal rat models; in rats with experimental NEC who did not receive melatonin, MDA and protein carbonyl content were higher and SOD and glutathione peroxidase levels were lower than in controls. On the contrary, rats with NEC who were also treated with melatonin had an OS profile similar to controls, suggesting a potential role of melatonin for reducing the severity of NEC [55].

### 5.4. Human Milk Oligosaccharides

HM oligosaccharides (HMO) have been shown to exert a series of beneficial effect on the neonatal gut [56]. Good et al. evaluated the role of 2′-fucosyllactose (HMO-2′FL), an abundant HMO, for the protection against NEC in rats. Their data showed that the addition of HMO-2′FL to milk formula reduced the severity of NEC. HMO-2′FL protective effects occurred via restoration of intestinal perfusion through upregulation of eNOS and downregulation of proinflammatory molecules including iNOS [57]. Thus, HMO may have indirect antioxidant properties by modulating the NO pathway. Interestingly, HMO do not seem to be affected by Holder pasteurization [43].

### 5.5. Lactoferrin

Lactoferrin plays a role in iron homeostasis, anti-inflammation, and host defense against microbial infections. Recently, it has been shown that lactoferrin inhibits the production of intracellular ROS in human mesenchymal stem cells, which suggests a potential role of lactoferrin in the prevention of OS-related damage [58]. Several RCTs have explored the effectiveness of oral lactoferrin in the prevention of NEC in preterm neonates. In a recent Cochrane review, results of six RCTs on this topic have been summarized; oral lactoferrin supplementation reduced late-onset sepsis and stage II and III NEC [59]. However, as the authors of the review also pointed out, the evidence about routine supplementation with lactoferrin is still of low quality and optimal dosing regimen, source of lactoferrin (human or bovine), long-term outcomes, and mechanisms are still to be clarified.

### 5.6. MicroRNAs

It has been recently documented that microRNAs (miRNAs) are present in HM and that specific miRNAs are involved in the innate and acquired immune response. Apparently, miRNA distribution and expression profile are not affected by pasteurization [60]. As for NEC, Ng et al. explored the role of dysregulated miRNAs in the pathogenesis of NEC and spontaneous intestinal perforation in preterm infants. According to the results of the study, dysregulation of miRNA/mRNA pairs played a significant role in the disease pathogenesis, with mechanisms also involving OS-related pathways [61]. This data warrant further exploration of HM miRNA characteristics and relation with neonatal gastrointestinal diseases.

### 5.7. Gut Microbiota

The role of intestinal dysbiosis is emerging as a major pathogenetic factor for NEC [62], and probiotics have been suggested as a promising intervention for preventing NEC in preterm infants [63]. It has been demonstrated that fecal microbiota transplantation (FMT) is effective in a mouse model of NEC through OS modulation and reduced TLR4-mediated colonic inflammation [23]. FMT eliminated superoxide production and promoted NO production, contrasting eNOS uncoupling. Furthermore, Ferretti et al. [20] identified iNOS as a potential mediator of the effects of both epithelial growth factor and indomethacin, which were, respectively, associated to positive and detrimental effects on the immature human gut. Their findings further indicate that the modulation of the NOS-NO pathway may represent a therapeutic opportunity for NEC.

## 6. Conclusions

NEC represents a clinical and research priority; a better understanding of NEC pathogenetic pathways may offer new treatment perspectives. OS has been shown to play a role in NEC cascade, and several antioxidant strategies have been explored. Despite promising preclinical studies, more clinical trials are needed. To date, the role of an exclusive HM diet is well recognized in the prevention of NEC. At the same time, it is known that DHM processing severely impairs HM functional properties, including defenses against OS. For this reason, future research should be targeted to explore in detail antioxidant properties of preterm and donor HM and to evaluate the best way to preserve or restore any deficient antioxidant function.
